# Evidence suggesting that oral corticosteroids increase mortality in stable chronic obstructive pulmonary disease

**DOI:** 10.1186/1465-9921-15-37

**Published:** 2014-04-03

**Authors:** Nobuyuki Horita, Naoki Miyazawa, Satoshi Morita, Ryota Kojima, Miyo Inoue, Yoshiaki Ishigatsubo, Takeshi Kaneko

**Affiliations:** 1 Department of Internal Medicine and Clinical Immunology, Yokohama City University Graduate School of Medicine, Yokohama, Japan; 2 Department of Respiratory Medicine, Saiseikai Yokohamashi Nanbu Hospital, Yokohama, Japan; 3 Department of Biostatistics and Epidemiology, Yokohama City University Medical Center, Yokohama, Japan; 4 Respiratory Disease Center, Yokohama City University Medical Center, Yokohama, Japan

## Abstract

**Background:**

Oral corticosteroids were used to control stable chronic obstructive pulmonary disease (COPD) decades ago. However, recent guidelines do not recommend long-term oral corticosteroids (LTOC) use for stable COPD patients, partly because it causes side-effects such as respiratory muscle deterioration and immunosuppression. Nonetheless, the impact of LTOC on life prognosis for stable COPD patients has not been clarified.

**Methods:**

We used the data of patients randomized to non-surgery treatment in the National Emphysema Treatment Trial. Severe and very severe stable COPD patients who were eligible for volume reduction surgery were recruited at 17 clinical centers in the United States and randomized during 1998-2002. Patients were followed-up for at least five years. Hazard ratios for death by LTOC were estimated by three models using Cox proportional hazard analysis and propensity score matching.

**Results:**

The pre-matching cohort comprised 444 patients (prescription of LTOC: 23.0%. Age: 66.6 ± 5.4 year old. Female: 35.6%. Percent predicted forced expiratory volume in one second: 27.0 ± 7.1%. Mortality during follow-up: 67.1%). Hazard ratio using a multiple-variable Cox model in the pre-matching cohort was 1.54 (P = 0.001). Propensity score matching was conducted with 26 parameters (C-statics: 0.73). The propensity-matched cohort comprised of 65 LTOC(+) cases and 195 LTOC(−) cases (prescription of LTOC: 25.0%. Age: 66.5 ± 5.3 year old. Female: 35.4%. Percent predicted forced expiratory volume in one second: 26.1 ± 6.8%. Mortality during follow-up: 71.3%). No parameters differed between cohorts. The hazard ratio using a single-variable Cox model in the propensity-score-matched cohort was 1.50 (P = 0.013). The hazard ratio using a multiple-variable Cox model in the propensity-score-matched cohort was 1.73 (P = 0.001).

**Conclusions:**

LTOC may increase the mortality of stable severe and very severe COPD patients.

## Background

In 1964, chronic obstructive pulmonary disease (COPD) was defined as a disease condition characterized by not reversible airflow limitation [1]. Since then, oral corticosteroids have often been used to control COPD [2]. In the 1980s, administration of 7.5 − 15 mg/day of long-term oral corticosteroids (LTOC) therapy was proved to improve the prognosis of patients with *chronic airflow obstruction*, a disease concept that partly overlaps with bronchial asthma and COPD [3,4]. A meta − analysis in 1991 revealed that oral corticosteroids improve forced expiratory volume in one second (FEV_1_) in stable COPD patients [5]. Various studies have repeatedly confirmed the favorable outcomes of therapy with systemic corticosteroids for acute exacerbation of COPD [6-10]. Guidelines published in 1995 thus indicated that LTOC may have beneficial effects for stable COPD patients [11].

However, other studies indicated that LTOC is potentially harmful for stable COPD patients because muscle strength and pulmonary function deteriorate after high dose of systemic corticosteroids [12], and because corticosteroids cause comorbidities such as diabetes, hypertension, and osteoporosis [13]. Furthermore, two prospective studies with a small number of patients reported that treatment of stable COPD patients with oral corticosteroids was not efficacious: in one study, two weeks of treatment with 40 mg of prednisone daily did not improve pulmonary symptoms or function [14]; in the other, a combination of inhaled plus oral corticosteroids for two years was not more effective than inhaled corticosteroids alone [15]. The current guidelines do not recommend LTOC for stable COPD patients [16]. In summary, many studies do not support the use of LTOC for stable COPD patients, but no study has clearly demonstrated a relationship between LTOC and life prognosis.

It is difficult to estimate how many patients worldwide is currently taking oral corticosteroids. But data from recently published randomized trial suggest that a considerable portion of patients with COPD is still taking oral corticosteroids. According to a report of the Understanding Potential Long-Term Impacts on Function with Tiotropium trial published in 2008, 8.4% of 5992 stable COPD cases took oral corticosteroids [17].

Given this background, it was not feasible to undertake a randomized controlled trial that evaluated the influence of LTOC on the life prognosis of stable COPD patients. Such a potentially harmful study is not ethically allowed. Even if feasible, evaluation of life prognosis demands the observation of a large number of patients for many years. Therefore, the aim of this study was to evaluate the life prognosis of patients treated with LTOC using the propensity score matching method.

## Methods

### Study design

**Figure 1:**
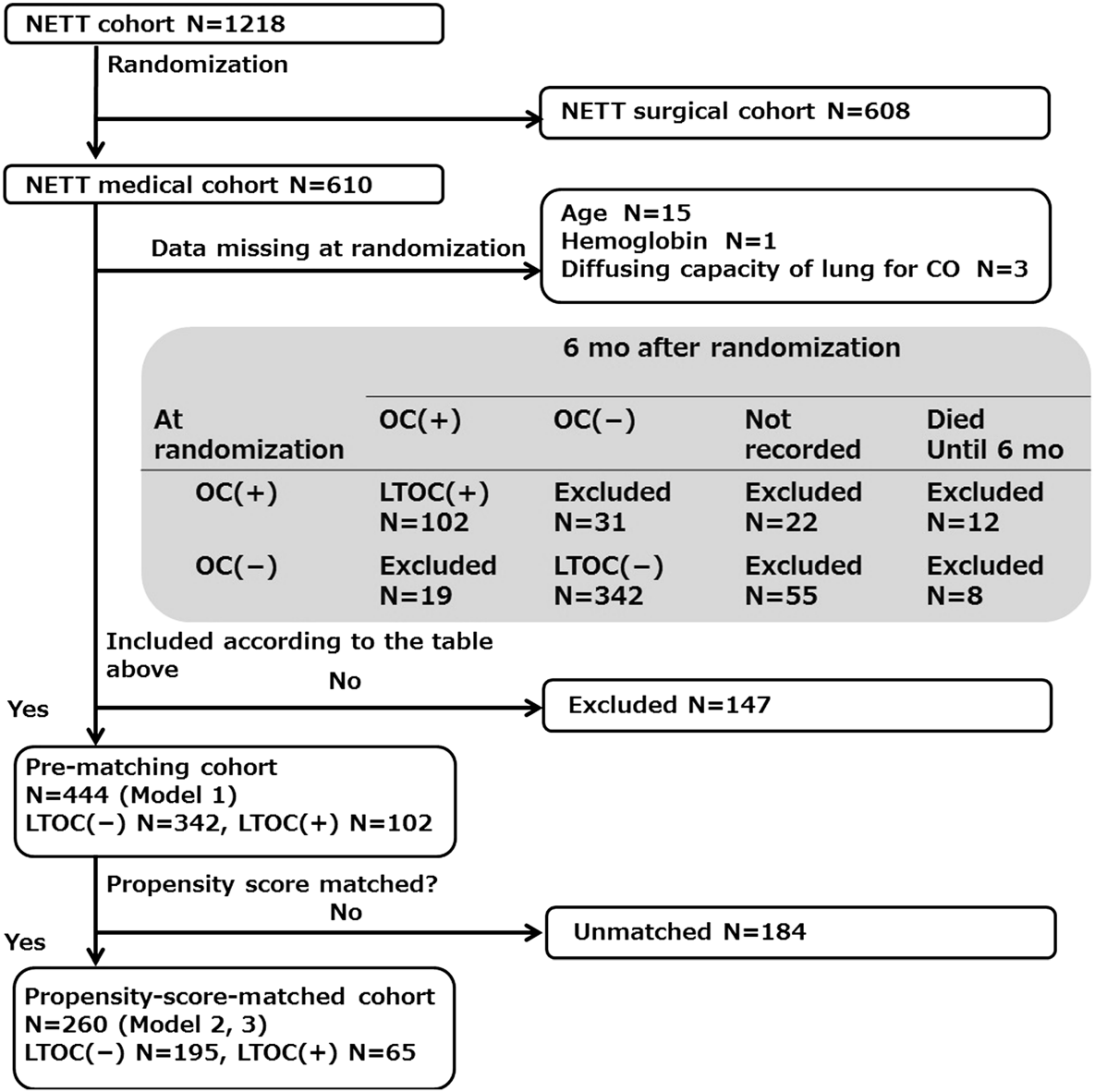
**Flow chart for patient entry.** NETT: National Emphysema Treatment Trial. N: Number of patients. OC: Oral corticosteroids prescription. LTOC: long-term oral corticosteroids.

The data set previously collected for the National Emphysema Treatment Trial (NETT) [17] was provided to us by the National Heart, Lung, and Blood Institute. We estimated the hazard ratio (HR) for death from LTOC in three Cox proportional hazards models. In Model 1, we calculated HR using a multiple-variable Cox model in a *pre-matching cohort*. Propensity-score matching was performed before the Model 2 and 3 analyses. In Model 2, we evaluated HR using a single-variable Cox model in a *propensity-score-matched cohort*. In Model 3, we estimated HR using a multiple-variable Cox model in the propensity-score-matched cohort (Figure 1). Our primary end point was death evaluated with HR by LTOC in the three models.

The data set previously collected for the National Emphysema Treatment Trial (NETT) was provided by the National Heart, Lung, and Blood Institute. The current study was approved by the Yokohama City Hospital Institutional Review Board. The need for informed consent was waived for this study due to patient anonymity and the observational nature of the study [18].

### Patient selection

The major entry criteria for the NETT study were the following for each patient: radiographic evidence of bilateral emphysema, % predicted FEV_1_ ≤ 45%, a pressure of carbon dioxide in artery (PaCO_2_) ≤ 60 mmHg, a pressure of oxygen in artery (PaO_2_) ≥ 45 mmHg, 6 − minute walking distance ≥ 140 m, participation in pulmonary rehabilitation, not at high risk for perioperative morbidity or mortality, suitable for lung volume reduction surgery, likely to complete the trial. Between January 1998 and July 2002, 3777 patients were evaluated in 17 clinical centers, and 1218 patients were eligible for randomization; 608 and 610 patients were randomly allocated to the surgical and medical cohort respectively. The criteria were described in greater detail in the previous report [18].

We used the data set of only the medical cohort patients for our study. Among 610 patients, patients for whom there were no data at baseline for age (N = 15), hemoglobin (N = 1), and diffusing capacity of the lungs for carbon monoxide (N = 3) were excluded from the study.

We defined an LTOC(+) case as a patient for whom oral corticosteroids were prescribed at both randomization and six months after randomization. We defined an LTOC(−) case likewise. Any patient who did not satisfy LTOC(+) and LTOC(−) definitions were excluded: (i) patients for whom oral corticosteroids were prescribed at either the randomization (N = 31) or six months after randomization (N = 19), (ii) patients for whom prescription of oral corticoid at six months after randomization was not checked (N = 77), (iii) patients who died within six months of randomization (N = 20). Finally, 342 LTOC(−) patients and 102 LTOC(+) patients were included in our study. These 444 patients comprised the *pre-matching cohort* in our study. Propensity score matching was performed for these 444 patients in a pre-matching cohort and the 260 matched patients comprised the *propensity-score-matched cohort* (Figure 1).

In our cohort, no death was observed in the six months following randomization because we excluded such patients. Therefore, our observation in this study starts six months after randomization.

### Treatments

The treatments were administered in close compliance with the guidelines [11]. The following treatments were administered by the primary care physician: smoking cessation, regular use of inhaled bronchodilators, oxygen therapy, influenza immunization, pneumococcal vaccination, pulmonary rehabilitation, and additional measures including oral corticosteroids. The details of the treatment methods are described in a previous report [18].

### Parameters selection and measurements

We selected the following factors for covariates: demographic factors, commonly used COPD parameters, factors related to acute exacerbation of COPD, and treatment (Table 1).

**Table 1:** Baseline characteristics of patients in the pre-matching cohort

	**All patients**	**Comparison of LTOC(+) and (−) cohorts**		
		**LTOC(+)**	**LTOC(−)**	**P**
**N**	**444**	**102**	**342**	
Age (year)	66.5 ± 5.4	66.6 ± 5.2	66.5 ± 5.4	0.636
Sex (female)	158 (35.6%)	35 (34.3%)	123 (36.0%)	0.760
Race (not white)	24 (5.4%)	8 (7.8%)	16 (4.7%)	0.215
Annual income < 30,000$	228 (51.4%)	53 (52%)	175 (51.2%)	0.888
% predicted FEV_1_	27.0 ± 7.1	25.2 ± 6.2	27.6 ± 7.2	0.004
FEV_1_ (L)	0.89 ± 0.25	0.73 ± 0.20	0.80 ± 0.26	0.004
FEV_1_/FVC (%)	31.1 ± 6.2	30.3 ± 6.2	31.4 ± 6.1	0.087
% predicted FVC	68.2 ± 15.4	65.5 ± 13.7	69.1 ± 15.7	0.051
FVC (L)	2.58 ± 0.82	2.47 ± 0.69	2.61 ± 8.3	0.200
Forced residual capacity (L)	6.00 ± 1.24	6.01 ± 1.20	6.00 ± 1.25	0.953
Hemoglobin adjusted DL_CO_	8.1 ± 3.1	7.6 ± 2.7	8.3 ± 3.2	0.057
Peak pulmonary artery pressure (mmHg)	33.7 ± 6.2	33.1 ± 6.1	33.8 ± 6.2	0.341
	(N = 377)	(N = 86)	(N = 291)	
Hemoglobin (g/dl)	14.4 ± 1.3	14.2 ± 1.3	14.5 ± 1.3	0.193
PaO_2_ (mmHg)	64.5 ± 10.0	63.4 ± 10.0	64.8 ± 10.0	0.224
PaCO_2_ (mmHg)	43.0 ± 5.8	43.4 ± 5.7	42.9 ± 5.8	0.326
Area of emphysema (%)	15.9 ± 10.1	14.9 ± 9.9	16.2 ± 10.2	0.299
	(N = 404)	(N = 96)	(N = 308)	
Body mass index (kg/m^2)	25.0 ± 3.5	24.4 ± 3.7	25.2 ± 3.4	0.051
Six − minute walk distance	375 ± 94	340 ± 86	386 ± 94	< 0.001
St. George Respiratory Questionnaire	53.0 ± 13.1	57.5 ± 12.4	51.7 ± 13.0	< 0.001
Shortness of Breath Questionnaire	62.2 ± 19.0	68.0 ± 17.4	60.5 ± 19.1	0.001
Beck Depression Inventory	9.1 ± 6.1	9.7 ± 5.9	9.0 ± 6.1	0.172
Recent emergency visit	77 (17.3%)	27 (26.5%)	50 (14.6%)	0.006
Recent hospital stay	56 (12.6%)	23 (22.5%)	33 (9.6%)	< 0.001
LTOT during sleep	290 (65.3%)	78 (76.5%)	212 (62.0%)	0.007
LTOT on exertion	300 (67.6%)	82 (80.4%)	218 (63.7%)	0.002
Inhaled corticosteroids	316 (71.2%)	76 (74.5%)	240 (70.2%)	0.396
Long acting beta agonist	197 (44.4%)	46 (45.1%)	151 (44.2%)	0.866
Short acting beta agonist	383 (86.3%)	92 (90.2%)	291 (85.1%)	0.188
Anticholinergic agent	347 (78.2%)	80 (78.4%)	267 (78.1%)	0.938
Theophylline	181 (40.8%)	49 (48.0%)	132 (38.6%)	0.089
Diuretics	56 (12.6%)	19 (18.6%)	37 (10.8%)	0.037

LTOC: long-term oral corticosteroids.

FEV_1_: forced expiratory volume in one second.

FVC: forced vital capacity.

DL_CO_: diffusing capacity of the lung for carbon monoxide.

PaO_2_: pressure of oxygen in artery.

PaCO_2_: pressure of carbon dioxide in artery.

LTOT: long term oxygen therapy.

Peak pulmonary artery pressure and area of emphysema were not evaluated for all patients due to lack of data.

Among these parameters, area of emphysematous change (%) and peak pulmonary artery pressure were not used for multiple-variable Cox model analysis and for generation of logistic regression formula for propensity score, as the data for some patients were not available. Forced vital capacity (FVC) (L), FEV_1_ (L), and FEV_1_/FVC (%) were also excluded from multiple-variable logistic regression to avoid possible multicollinearity with % predicted FVC and/or % predicted FEV_1_. Other 26 parameter were included for multi-variable logistic regression.

Spirometric data were collected after bronchodilator use. The diffusing capacity of the lungs for carbon monoxide was adjusted by hemoglobin: the diffusing capacity of the lungs for carbon monoxide × hemoglobin / 0.0697. PaO_2_ and PaCO_2_ were measured in ambient air. An area of emphysema was an area below −950 Hounsfield. The peak pulmonary artery pressure was measured using an echocardiogram or right heart catheterization. The peak pulmonary artery pressure by echocardiogram was estimated as the “mean right atrial pressure + 4 × (estimated tricuspid peak systolic velocity)^2^. ” A recent emergency hospital stay included both admission and overstay in an acute care facility during the three months preceding randomization. Death was defined as death from all causes, not only respiratory related death. The details of the measurement methods have been discussed in the previous report [18].

### Prescription of oral corticosteroids during follow-up

We checked whether oral corticosteroids were prescribed for patients during observation to discover how patients “compliant” were to allocated treatment arms, LTOC(+) or LTOC(−). All analyses evaluating death were performed independently of oral corticosteroids prescription during the observation, i.e. the intention-to-treat principle was followed.

### Analysis

A Wilcoxon rank sum test and a Chi − square test (Yates’s corrected, if necessary) were used to compare the two cohorts. A Cox proportional hazard model was used to evaluate life prognosis. The inclusion and exclusion criteria of P = 0.1 were used for stepwise variable selection for a multiple-variable Cox proportional hazard model. A Kaplan − Meier curve and a Log-rank test were also used for comparison of life prognosis.

Using the propensity score based on 26 parameters, neighborhood propensity score matching [19] was performed with a maximal distance of 0.03 in propensity score. All 444 patients in pre-matching cohort were included for the matching process. One LTOC(+) case was matched with three LTOC(−) controls because the pre-matching cohort contained 3.3 (= 342/102) times as many LTOC(−) patients as LTOC(+) patients. The quality of matching was evaluated by C − statistics and by comparing patient characteristics between cohorts.

## Results and discussion

### Pre-matching cohort

**Figure 2:**
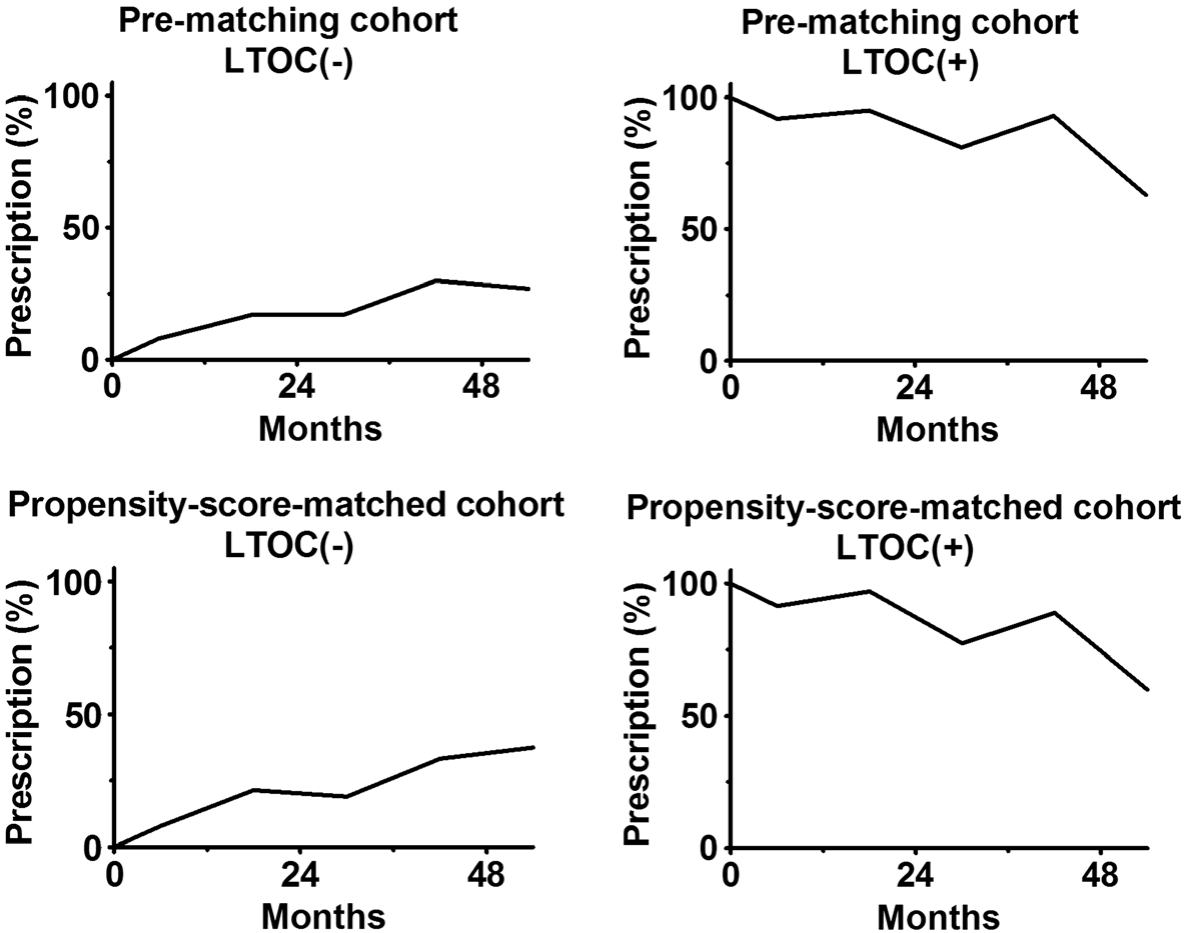
Prescription of oral corticosteroids during follow-up.

The pre-matching cohort included 444 patients, whose mean age was 66.5 ± 5.4 years and the mean % predicted FEV_1_ was 27.0 ± 7.1% at randomization. Of 444 patients, 158 (35.6%) were women, 102 (23.0%) were LTOC(+) cases, and 342 (67.0%) were LTOC(−) cases. Some measurements namely % predicted FEV_1_, six-minute walking distance, St. George Respiratory Questionnaire, Shortness of Breath Questionnaire, emergency visit, and emergency hospital stay indicated that patients with LTOC were generally in poorer condition among LTOC(+) patients (Table 1). During the observation, 298 patients (67.1%) died. No patient was censored before the 1949^th^ day. Oral corticosteroids were prescribed for no more than 30% of LTOC(−) patients and no less than 63% of LTOC(+) patients until 54 mo follow-up (Figure 2).

Although all patients in the cohort had substantially advanced COPD, the prescription rates of some medications, especially long-acting beta agonist (44.4%) and theophylline (40.8%), were relatively low, considering the current guidelines that strongly recommend use of bronchodilators. It is probably because the cohort was recruited since 1997 and the guideline in this era [11] did not highly appreciate these medications as the current guidelines do.

### Model 1

**Figure 3:**
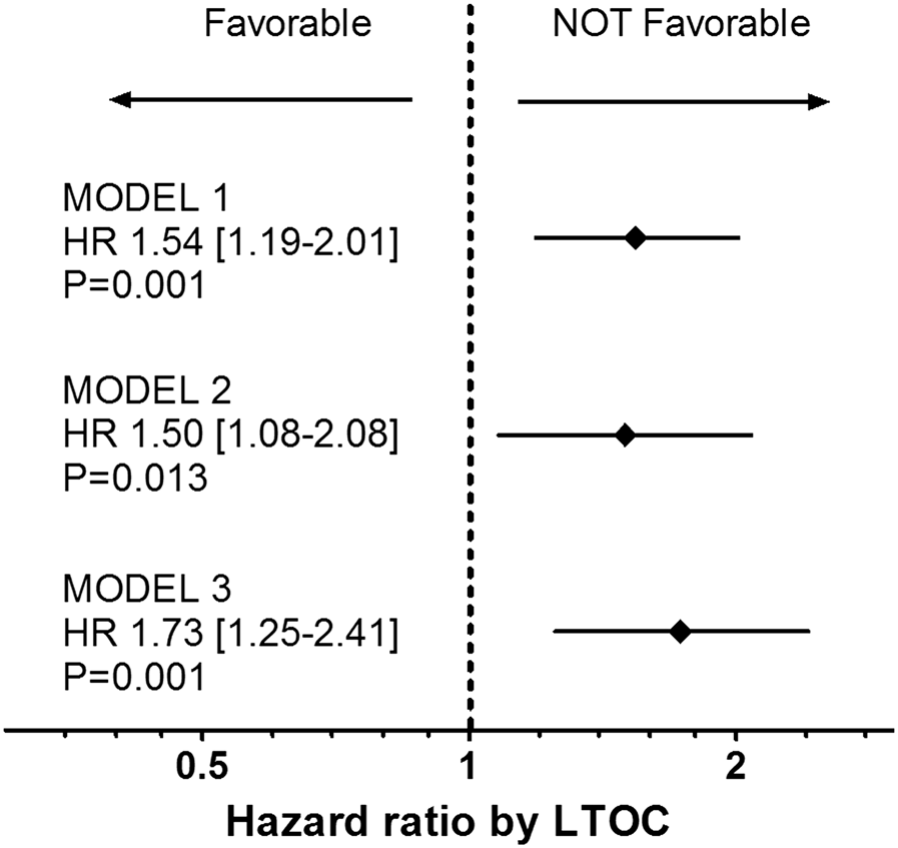
**Hazard ratio for death by long-term oral corticosteroids (LTOC) treatment.** Model 1: multiple-variable Cox model in pre-matching cohort. Model 2: single-variable Cox model in the propensity-score-matched cohort. Model 3: multiple-variable Cox model in the propensity-score-matched cohort. [ ]: 95%CI.

The stepwise multiple-variable Cox model analysis, which initially included LTOC and 26 other coverables as independent variable candidates, was performed in the pre-matching cohort. Nine independent variables including LTOC remained in the last model. The HR for death from LTOC was 1.54 (95%CI: 1.19 − 2.01. P = 0.001) (Figure 3).

### Propensity-score matching

For propensity-score matching, data of all 444 patients in pre-matching cohort were used (Figure 1). Logistic regression formula was generated using 26 parameters. The body mass index, six − minute walk distance, and St. George’s Respiratory Questionnaire were significantly related to LTOC therapy in the logistic regression analysis for propensity matching. The mean propensity scores among LTOC(−) and LTOC(+) patients were 0.20 ± 0.13 and 0.33 ± 0.17 respectively. The C − statistic was 0.73. Sixty-five LTOC(+) patients were matched with 195 LTOC(−) patients. The propensity-score-matched cohort included 260 patients (Figure 1), whose mean age was 66.5 ± 5.3 years and the mean % predicted FEV_1_ was 26.1 ± 6.8%. Of 280 patients, 92 (35.4%) were women (Table 2). No measurement showed a significant difference between cohorts (Table 2). Proportions of oral corticosteroids prescription in the propensity-score-matched cohort were similar to those in the pre-matched cohorts (Figure 2).

**Table 2:** Baseline characteristics of patients in the propensity-score-matched cohort

	**LTOC(+)**	**LTOC(−)**	
	**65**	**195**	**P**
Age (year)	66.3 ± 5.0	66.6 ± 5.3	0.295
Sex (female)	23 (35.4%)	69 (35.4%)	1
Race (not white)	3 (4.6%)	10 (5.1%)	0.870
Annual income < 30,000$	36 (55.4%)	100 (51.3%)	0.566
% predicted FEV_1_	26.4 ± 6.8	26.0 ± 6.6	0.461
[FEV_1_ (L)]	0.78 ± 0.22	0.76 ± 0.25	0.294
[FEV_1_/FVC (%)]	30.9 ± 5.6	31.0 ± 6.0	0.746
% predicted FVC	66.9 ± 13.8	66.1 ± 14.6	0.653
[FVC (L)]	2.53 ± 0.64	2.51 ± 0.78	0.644
Forced residual capacity (L)	6.05 ± 1.22	6.05 ± 1.24	0.986
Hemoglobin adjusted DL_CO_	7.8 ± 3.0	7.9 ± 3.0	0.733
[Peak Pulmonary Artery Pressure (mmHg)]	33.8 ± 6.0	34.0 ± 6.6	0.782
	(N = 55)	(N = 167)	
Hemoglobin (g/dl)	14.4 ± 1.1	14.3 ± 1.3	0.676
PaO_2_ (mmHg)	63.3 ± 10.2	63.3 ± 9.8	0.989
PaCO_2_ (mmHg)	43.4 ± 5.7	43.7 ± 6.2	0.679
[Area of emphysema (%)]	13.5 ± 9.4	15.9 ± 9.8	0.085
	(N = 60)	(N = 173)	
Body mass index (kg/m^2)	24.7 ± 3.6	24.6 ± 3.5	0.791
Six − minute walk distance	362 ± 79	366 ± 90	0.689
St. George Respiratory Questionnaire	53.8 ± 11.8	53.9 ± 13.3	0.892
Shortness of Breath Questionnaire	64.0 ± 16.4	63.8 ± 18.9	0.915
Beck Depression Inventory	9.0 ± 5.9	9.1 ± 6.3	0.988
Recent emergency visit	13 (20.0%)	34 (17.4%)	0.642
Recent hospital stay	9 (13.9%)	24 (12.3%)	0.747
LTOT during sleep	48 (73.8%)	144 (73.8%)	1
LTOT on exertion	50 (76.9%)	155 (79.5%)	0.661
Inhaled corticosteroids	49 (75.4%)	140 (71.8%)	0.574
Long acting beta agonist	31 (47.7%)	94 (48.2%)	0.942
Short acting beta agonist	57 (87.7%)	167 (85.6%)	0.678
Anticholinergic agent	49 (75.4%)	155 (79.5%)	0.486
Theophylline	30 (46.2%)	91 (46.7%)	0.943
Diuretics	11 (16.9%)	31 (15.9%)	0.846

[Parameters]: parameters that were not used in multi-variable logistic regression analysis for Cox model and propensity score matching were shown in brackets.

LTOC: long-term oral corticosteroids.

FEV_1_: forced expiratory volume in one second.

FVC: forced vital capacity.

DL_CO_: diffusing capacity of the lung for carbon monoxide.

PaO_2_: pressure of oxygen in artery.

PaCO_2_: pressure of carbon dioxide in artery.

LTOT: long term oxygen therapy.

Peak pulmonary artery pressure and area of emphysema were not evaluated for all patients due to lack of data.

### Model 2

**Figure 4:**
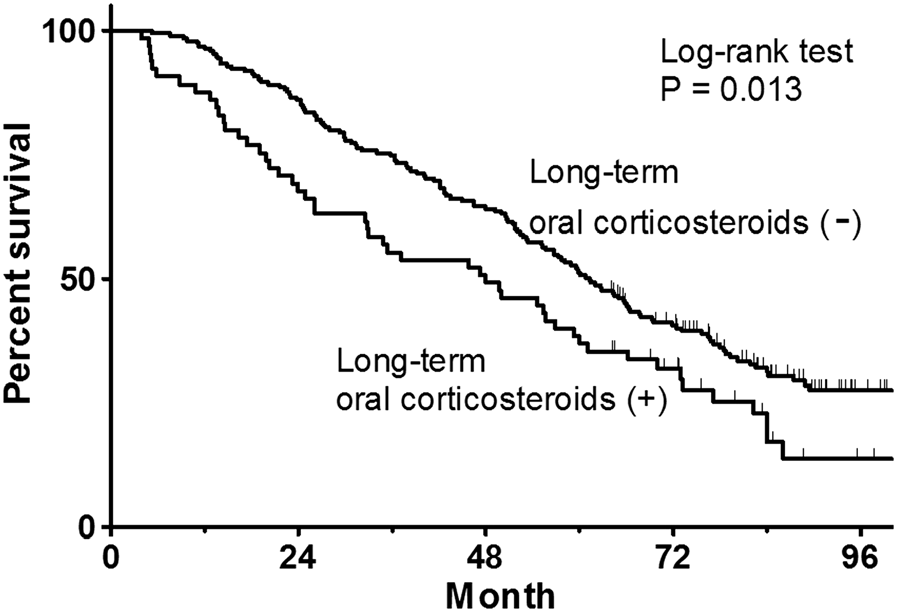
Kaplan-Meier survival curve on the propensity-score-matched cohort (Model 2).

In the propensity-score-matched cohort, a single-variable Cox model analysis revealed that HR for death by LTOC was 1.50 (95%CI: 1.08 − 2.08, P = 0.013) (Figure 3). The Kaplan-Meier curve also showed that LTOC(+) patients had poorer survival prognosis than LTOC(−) patients (P = 0.013, Log-rank test) (Figure 4).

### Model 3

A stepwise multiple-variable Cox model analysis which initially included LTOC and 26 other coverables as independent variable candidates was performed in the propensity-score-matched cohort. Nine independent variables including LTOC remained in the last model. The HR for death from LTOC was 1.73 (95%CI: 1.25 − 2.41. P = 0.001) (Figure 3).

## Discussion

To our knowledge, this is the first study to evaluate the effect of oral corticosteroids on the long-term life prognosis of COPD patients in solid manner. The HR for death from LTOC estimated in the three models ranged from 1.50 to 1.73. LTOCs were generally prescribed for patients in a deteriorated condition (Table 1), as suggested in the old guideline [11]. However, LTOC further deteriorated the life prognosis of these patients. We still believe that systemic corticosteroids are effective for acute exacerbation of COPD [6-10]. However, it should be discontinued after the acute phase. Discontinuation of LTOC has already been proven to be safe [20]. We strongly support the current guidelines which do not recommend LTOC for stable COPD patients [16].

One of the most important side effect of LTOC is respiratory muscle deterioration. Decramer observed 21 patients with COPD or asthma who were admitted to hospitals due to exacerbations [12]. The average daily dose of corticosteroids taken in the previous six months was significantly related to inspiratory and expiratory muscle strength. These relationships were independent of the degree of bronchial obstruction estimated by % predicted FEV_1_[12]. In addition, glucocorticoids, which are the most potent anti-inflammatory and immunosuppressive agents, inhibit synthesis of almost all known cytokines and of several cell surface molecules required for immune function [21]. It is easily anticipated that patients who is taking oral corticosteroids have more chance to suffer from potentially life-threatening acute exacerbation due to the immunosuppressive states.

The results of some previous studies may appear to conflict with the current study [3-5]. Postma observed 79 patients with chronic airflow obstruction, i.e. COPD or asthma, whose FEV_1_ was less than 1000 ml for more than 14 years [3]. The same author also observed 139 less severe patients with chronic airflow obstruction whose FEV_1_ was more than 1200 ml for more 11 years on average [4]. Both studies concluded that oral prednisolone, in doses above 7.5 mg/d, may slow down the deterioration of FEV_1_[3,4]. We should interpret the results carefully, because Postma’s study observed COPD patients together with asthma patients. Considering Decramer’s study [12] and Postma’s study [3,4], LTOC may improve the respiratory function of asthma patients but may worsen that of COPD patients.

Callahan conducted a meta-analysis to evaluate oral corticosteroids therapy for patients with stable COPD [5]. He scrutinized 10 placebo controlled randomized controlled trials and concluded that patients with stable COPD receiving oral corticosteroids therapy have a 20% or greater improvement in baseline FEV_1_ approximately 10% more often than similar patients receiving a placebo. The most important difference between Callahan’s meta-analysis and Decramer’s study [12] is the duration of observation. Among 10 randomized controlled trials in the meta-analysis, nine observed COPD patients for 14 days or less, and one observed COPD patients for eight weeks [5]. Oral corticosteroids may improve FEV_1_ of COPD patients if prescribed for weeks. However, it may deteriorate FEV_1_, if prescribed for months or years. Another considerable limitation of Callahan’s study is a publication bias, which has a considerable impact on the result of the meta-analysis [5].

Our study had several limitations. First, it was an observational study, and not a randomized controlled trial. Since the current guidelines [16] do not recommend administering LTOC, a randomized controlled trial was not thought to be a feasible design. Our study design was the next best to a randomized controlled trial. Second, the dosage of oral corticosteroids in this study was not verified. Considering that the long − term dosages in previous studies were 5 mg/d [15], 7.5 mg/d [3], and 10 − 15 mg/d [4], a similar dosage might be prescribed for patients in our cohort. Third, our cohort contained only patients whose % predicted FEV_1_ ≤ 45% and this study did not explain whether LTOC worsens the life prognosis of COPD patients with % predicted FEV_1_ > 45. However, LTOC had been already regarded as the last option for patients with uncontrolled advanced COPD [11]. There was no reason to recommend LTOC for patients with mild or moderate COPD. Together with other inclusion and exclusion criteria, the current cohort may have slight difference from “real-world” patients. Thus, we should interpret the result with caution. Fourth, as in other observational studies, oral corticosteroids before observation may cause some bias. Oral corticosteroids administered before randomization probably deteriorate baseline characteristics of LTOC(+) patients [12,13]. But the LTOC(+) and (−) patients in the propensity-matched cohort were nearly equal (Table 2) by cancelling the harm from oral corticosteroids before randomization. The matched LTOC(+) cases would have better condition, if they had not been treated with oral corticosteroids. Nonetheless, they had poorer life prognosis than LTOC(−) cases. This bias made true impact of medication seem smaller. Fifth, it is not fully confirmed that LTOC(+) patients actually took oral corticosteroids during the entire follow-up period. The definition of LTOC(+) patients in the current study might reflect repeated short course oral corticosteroid. In addition, patient’s adherence to prescribed medication is not evaluated. This bias shift the observed HR toward the null, as drop-out cases in intentional-to-treat analysis do. Thus, oral corticosteroids may have stronger impact on mortality than we estimated in this study.

## Conclusion

The HR for mortality from LTOC among COPD patients with % predicted FEV_1_ < 45% calculated with the Cox proportional hazard model ranged from 1.50 to 1.73. We do not recommend oral corticosteroids treatment for patients with stable COPD.

## Abbreviations

COPD: Chronic obstructive pulmonary disease; LTOC: Long-term oral corticosteroids; HR: Hazard ratio; FEV1: Forced expiratory volume in one second; FVC: Forced vital capacity; PaO2: Pressure of oxygen in artery; PaCO2: Pressure of carbon dioxide in artery; NETT: National Emphysema Treatment Trial.

## Competing interests

None of the investigators declare any real or perceived conflicts of interest pertaining to the subject of this manuscript.

## Authors’ contributions

All authors contributed conception, design, analysis, interpretation, drafting, revising, and final approval of the manuscript. NH served as a principal investigator. NM, RK and MI mainly provided analysis and drafting. SM especially worked as statistician. YI and TK especially provided conception and revising.

## Financial statement

No support in the form of grants, gifts, equipment, and/or drugs was provided.
